# Molecular detection of viruses in Kenyan bats and discovery of novel astroviruses, caliciviruses and rotaviruses

**DOI:** 10.1007/s12250-016-3930-2

**Published:** 2017-04-06

**Authors:** Cecilia Waruhiu, Sheila Ommeh, Vincent Obanda, Bernard Agwanda, Francis Gakuya, Xing-Yi Ge, Xing-Lou Yang, Li-Jun Wu, Ali Zohaib, Ben Hu, Zheng-Li Shi

**Affiliations:** 1 Key Lab of Special Pathogens, Wuhan Institute of Virology, Chinese Academy of Sciences, Wuhan, 430071 China; 2 Sino-Africa Joint Research Center, Chinese Academy of Sciences, Wuhan, 430071 China; 3 Institute of Biotechnology Research, Jomo Kenyatta University of Science and Technology, Nairobi, 00200 Kenya; 4 Veterinary Services Department, Kenya Wildlife Service, Nairobi, 00100 Kenya; 5 Mammalogy Section, National Museum of Kenya, Nairobi, 00100 Kenya

**Keywords:** astroviruses (AstVs), calicivirus (CalVs), *Rotavirus A*, 229-E-like bat coronavirus

## Abstract

This is the first country-wide surveillance of bat-borne viruses in Kenya spanning from 2012–2015 covering sites perceived to have medium to high level bat-human interaction. The objective of this surveillance study was to apply a non-invasive approach using fresh feces to detect viruses circulating within the diverse species of Kenyan bats. We screened for both DNA and RNA viruses; specifically, astroviruses (AstVs), adenoviruses (ADVs), caliciviruses (CalVs), coronaviruses (CoVs), flaviviruses, filoviruses, paramyxoviruses (PMVs), polyomaviruses (PYVs) and rotaviruses. We used family-specific primers, amplicon sequencing and further characterization by phylogenetic analysis. Except for filoviruses, eight virus families were detected with varying distributions and positive rates across the five regions (former provinces) studied. AstVs (12.83%), CoVs (3.97%), PMV (2.4%), ADV (2.26%), PYV (1.65%), CalVs (0.29%), rotavirus (0.19%) and flavivirus (0.19%). Novel CalVs were detected in *Rousettus aegyptiacus* and *Mops condylurus* while novel Rotavirus-A-related viruses were detected in *Taphozous* bats and *R. aegyptiacus*. The two *Rotavirus A* (RVA) strains detected were highly related to human strains with VP6 genotypes I2 and I16. Genotype I16 has previously been assigned to human RVA-strain B10 from Kenya only, which raises public health concern, particularly considering increased human-bat interaction. Additionally, 229E-like bat CoVs were detected in samples originating from *Hipposideros* bats roosting in sites with high human activity. Our findings confirm the presence of diverse viruses in Kenyan bats while providing extended knowledge on bat virus distribution. The detection of viruses highly related to human strains and hence of public health concern, underscores the importance of continuous surveillance.

**Electronic Supplementary Material:**

Supplementary material is available for this article at 10.1007/s12250-016-3930-2 and is accessible for authorized users.

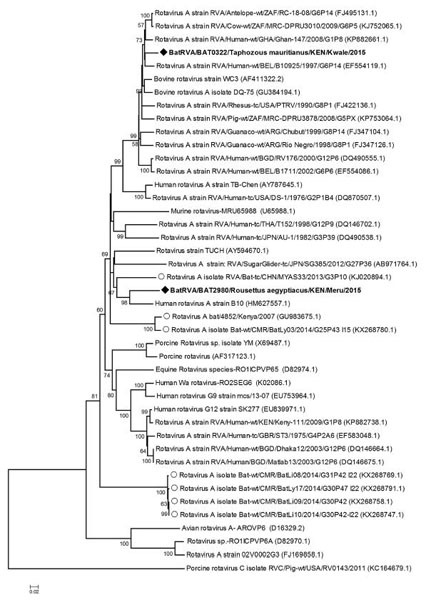

### INTRODUCTION

Bats (Order *Chiroptera*) are a group of highly diverse mammalian species that occurs throughout the world and accounts for more than 25% of mammals (Olival, 2016). Bats occur in large colonies and have evolved unique adaptations such as flight (O'Shea et al., 2014) which allow them to travel over long distances. Over time, bats have extended their roost sites from forested areas, caves and other isolated sites to habitats with substantial human activity. This can be attributed to anthropogenic activities such as urbanization and deforestation that have resulted in destruction of natural bat habitats (Plowright et al., 2015).

In the last decade, bats have been recognized as an important natural reservoir for zoonotic viruses affecting both humans and livestock (Plowright et al., 2015). Both RNA and DNA virus families have been detected in both frugivorous and insectivorous bats including RNA viruses: coronaviruses, filoviruses, lyssaviruses, paramyxoviruses, flaviviruses, astroviruses, caliciviruses; DNA viruses: adenoviruses, herpesviruses, poxviruses, polyomaviruses and papillomavirus among others (Shi, 2013). Over 200 virus species have been reported in bats (Moratelli et al., 2015) and additional novel viruses continue to be detected, yet the global diversity of viruses in bats is still thought to be largely unknown (Anthony et al., 2013). In many instances, some of the viruses harbored in bats such as; Nipah virus, severe acute respiratory syndrome coronavirus (SARS-CoV), multiple strains of lyssavirus, Marburg virus and Ebola virus have spilled over to humans and livestock causing severe disease in different parts of the world (Chua et al., 2000; Leroy et al., 2005; Wang et al., 2005).

Africa is one of the major hotspots of emerging infectious diseases (Jones et al., 2008) hence signifying need for enhance surveillance efforts as well as conservation efforts aimed at mitigating the risks of zoonotic disease emergence. Studies conducted in African countries such as Ghana, Gabon, Cameroon among others have reported circulation of several virus families in bats, such as; *Filoviridae*, *Coronaviridae*, *Reoviridae*, *Paramyxoviridae*, *Adenoviridae* and *Polyomaviridae* (Baker et al., 2013; Corman et al., 2015; Drexler et al., 2009; Yinda et al., 2016). These findings, as well as occurrence of outbreaks linked to bats, such as Ebola virus disease, affirm the recommendation of Jones *et al.* (2008), on the necessity for targeted research in Africa as one of the high-risk zones for emergence of zoonotic pathogens (Gatherer, 2014; Marí Saéz et al., 2014). Information of the geographical distribution and genetic diversity of viruses harbored by bats in Kenya is quite limited, yet bat is one of the mammalian orders associated with a growing list of deadly emerging pathogens. Although there have been some studies on bat viruses carried out between 2007 to 2011, they are insufficient to display the epidemiological status of bat viruses in Kenya (Kading et al., 2013; Kuzmin et al., 2010a; Kuzmin et al., 2010b; Tao et al., 2013; Tong et al., 2009). As early as 1982, Marburg virus (MARV) had been linked to the death of a febrile ill patient in Kenya (Smith et al., 1982) and a similar fatal case of MARV infection occurred a few years later in 1987 (Johnson et al., 1996) was linked to a visit to a bat cave. In 2007, MARV was detected in tissue of Egyptian fruit bat *Rousettus aegyptiacus* (Kuzmin, et al., 2010). Notably, the MARV strain detected by Kuzmin (2010) differed genetically from ones reported in 1980 and 1987 suggesting circulation of several strains in the same cave and/or its environments. In a more significant case, a close encounter with a bat was reported to have resulted in the death of a tourist due to infection with Duvenhage virus, a bat rabies virus (Van Thiel et al., 2009), presenting evidence of direct virus transmission from bats to humans.

An overarching quest in virus discovery is to detect viruses and also determine transmission pathways especially to humans. Bats are likely to shed some viruses through feces and urine, materials that can be highly infectious to humans. Therefore, this study focused on collecting freshly voided bat feces for molecular detection of multiple virus families. Detection of the virus in these samples, will further strengthen the recommended use of non-invasive methods for virus discovery thereby furthering conservation efforts. In addition, presence of viruses in bat feces reiterates the risk of virus transmission to humans, especially through direct hand contact with freshly voided feces/urine. These factors are important especially in understanding the epidemiology of bat-borne viruses as humans increase their encroachment to wildlife habitats in most African countries including Kenya. In this study, we reveal the virus diversity from bat fecal samples collected in different habitats and in different geographical locations of Kenya.

### MATERIALS AND METHODS

#### Study area and sample collection

Fresh bat feces were collected from 48 sites within 17 counties in 5-formerprovincial regions of Kenya (Figure 1) within the period of November 2012 to November 2015. The sampled bat colonies included roosts in caves, trees, inhabited and abandoned buildings (offices and homes) all with relatively high level of human-bat interaction. Fresh fecal samples (One fecal pellet per tube) were collected and placed in RNALater preservative solution (QIAGEN, Germany) as described previously (Ge et al., 2011). Briefly, to collect the fresh fecal pellets, clean polythene sheets measuring 2.0×2.0 m were spread under known bat roosting sites at about 18:00 hours and left to remain until the next morning at about 5:30–6:00 hours the next morning when the pellets were picked into vials that were placed in cooled iceboxes. Samples were transported to the laboratory and stored –80℃ until they were analyzed. Bat species composition in particular roost sites were identified by an experienced chiropterologist and further classified by mitochondrion

Figure 1: Map showing bat sample collection sites in Kenya within 2012–2015. Schematic map of Kenya showing bat sampling locations. Each pin ( ) represents the GPS mapping of a single or multiple sampling sites. Samples were collected within 2012–2015 in 17 counties, within 5-former provincial regions, outlined in the pin label (C-Coast, E-Eastern, R-Rift Valley, N-Nyanza and W-Western) and 48 individual sites.

*cytochrome b* amplification and sequencing.

### Sample processing and Virus detection

The samples were completely homogenized by vortexing for 15 seconds and then centrifuged at 12000g for 1 minute to obtain a clear supernatant. Viral nucleic acid was extracted from 200μL of the supernatant using the high pure Viral RNA kit (Roche, Germany) following manufacturer's instructions. Virus screening was performed by RT-PCR using the Invitrogen OneStep-RT PCR kit using gene specific primers (Supplementary Table S1). Virus families screened included *Coronaviridae* (CoV), *Filoviridae*, *Reoviridae*, *Astroviridae* (AstV), *Adenoviridae* (ADV), *Polyomaviridae* (PYV), *Paramyxoviridae* (PMV) and *Caliciviridae* (CalV). The RT-PCR was performed in a 25 μL reaction mix containing 12.5 μL PCR 2 × reaction mix Buffer, 10pmol of each primer, 2.5 mol/L $MgSO_4$, 20 U RNase Inhibitor, 1 μL SuperScript III/ Platinum Taq Enzyme Mix, and 5 μL RNA. A nested/ hemi-nested PCR was done using 1uL of the first-round PCR product as a template in a 25 μL reaction mix contained 2.5 μL PCR reaction buffer, 5 pmol of each primer, 50 mmol/L $MgCl_2$, 0.5 mmol/L dNTP and 0.1 μL Platinum Taq Enzyme (Invitrogen). The PCR products were visualized on 1% agarose gel after electrophoresis. The amplicons were gel purified and sequenced directly using the ABI prism sequencer or cloned using pGEM-T Easy Vector System before sequencing.

### Sequence and phylogenetic analysis

Sequences were edited using Bio-edit program and preliminary identification done by GenBank Blast searches on the non-redundant database. Sequences were aligned using ClustalW and phylogenetic trees constructed using the maximum likelihood method with an appropriate model and 1000 bootstrap replications. Phylogenetic analysis using MEGA v5.0 (Tamura et al., 2011) software was carried out with representative sequences of the related viral families available in public databases.

## RESULTS

A total of 3334 bat fecal samples were collected from various sites in 17 counties in Kenya between 2012 and 2015 (Figure 1) and preserved in RNALater solution. We identified 8 virus families including; *Astroviridae*, *Adenoviridae*, *Caliciviridae*, *Coronaviridae*, *Flaviviridae*, *Paramyxoviridae*, *Polyomaviridae* and *Reoviridae* (Table 1). We did not detect filoviruses in any of the samples. The Coastal region of Kenya had the richest diversity since all the 8 virus families were detected. Virus families CoVs, AstVs, ADVs and PMVs were most spatially distributed as they occurred in all the 5-former provincial regions. For the first time in Kenya, AstVs and CalVs were detected in bats, while new genotypes of RVA are reported in *R. aegyptiacus* and *T. mauritianus*.

Table 1. Results summary of virus screening in Kenyan bat fecal samples

| Locations (County) | Bat species sampled in County | Astroviruses | | Calicivirus | | Coronavirus | | Paramyxovirus | | Rotavirus | |
|---|---|---|---|---|---|---|---|---|---|---|---|
| | | +ve/ Tested | Species positive | +ve/ Tested | Species positive | +ve/ Tested | Species positive | +ve/ Tested | Species positive | +ve/ Tested | Species positive |
| Kilifi | *C. cor*, *E. helvum*, *R. aegyptiacus*, *Rh. fumigatus*, *M. minor*, *C. afra*, *Tr. affer*, *H. caffer*, *Ch. pumilus*. | 25/156 | *E. helvum* (9), *C. cor* (7), *R. aegyptiacus* (6), *T. affer* (2), *M. minor* (1). | 0/156 | | 6/226 | *E. helvum* | 2/360 | *E. helvum* | 0/156 | |
| Kwale | *R. aegyptiacus*, *Rh. fumigatus*, *Ta. mauritianus.*, *H. vittatus*, *H. gigas*, *H. commersonii*, *C. afra*, *M. minor.* | 23/95 | *T. Mauritianus* (5), *H. commersonii* (7), *C. afra* (9), *M. minor* (2) | 1/95 | *R. aegyptiacus* | 9/175 | *R. aegyptiacus* | 19/384 | *T. Mauritianus* (3), *C. afra* (6), *H. vittatus* (10) | 1/95 | *T. mauritianus* |
| Mombasa | *E. helvum* | 0/10 | | 0/10 | | 1/14 | *E. helvum* | 0/24 | | 0/10 | |
| Taita Taveta | *H. caffer*, *Ep. minimus.* | 0/20 | | 0/20 | | 2/33 | *H. caffer* | 2/43 | *H. caffer* | 0/20 | |
| Baringo | *Ep. minor*, *M. condylurus*, *Ch. pumulis.* | 0/20 | | 0/20 | | 0/45 | | 1/45 | *M. condylurus* | 0/20 | |
| Kericho | *Rh. eloquens*, *C. arfa*, *M. minor*, *H. ruber.* | 5/13 | *M. minor* | 0/13 | | 5/44 | *M. minor* | 0/44 | | 0/13 | |
| Migori | *T. aegyptiaca*, *Ch. Pumilus*, *C. afra*, *Rh. fumigatus*, *M. condylurus* | 7/75 | *C. afra* (4), *Ch. pumilus* (3) | 0/75 | | 6/180 | *M. condylurus* (1), *Ch. Pumilus* (5) | 0/180 | | 0/75 | |
| Kajiado | *O. martiensseni* | 0/60 | | 0/60 | | 22/150 | | 25/150 | | 0/60 | |
| Kisii | *E. helvum* | 9/135 | | 0/135 | | 20/185 | | 1/185 | | 0/135 | |
| Busia | *C. afra*, *Rh. landeri*, *M. condylurus*, *Ch. pumilus* | 14/155 | *C. afra* (12), *Ch. pumilus.* (1), *M. condylurus* (1) | 0/155 | | 3/230 | *M. condylurus* (2), *Rh. Landeri* (1) | 3/230 | *C. afra* | 0/155 | |
| Vihiga | *E. helvum* | 0/10 | | 0/10 | | 1/25 | | 0/25 | | 0/10 | |
| Kisumu | *E. helvum* | 0/15 | | 0/15 | | 0/45 | | 0/45 | | 0/15 | |
| Trans-Nzoia | *R. aegyptiacus* | 5/35 | *R. aegyptiacus* | 0/35 | | 0/75 | | 0/75 | | 0/35 | |
| Nakuru | *M. minor*, *My. tricolor*, *Rh. fumigatus*, *C. afra*, *H. caffer.* | 24/40 | *M. minor* (22), *My. tricolor* (2) | 0/40 | | 0/115 | | 3/115 | *M. minor* | 0/40 | |
| Marsabit | *M. condylurus*, *T. aegyptiaca*, *Ch. pumilus* | 0/40 | | 2/40 | *M. condylurus* | 0/95 | | 0/95 | | 0/40 | |
| Kitui | *Rh. fumigatus*, *H. caffer*, *M. condylurus*, *T. aegyptiaca*, *Ch. pumilus.* | 0/48 | | 0/48 | | 2/107 | *Rh. Fumigatus* (1), *H. caffer* (1) | 1/107 | *M. condylurus* | 0/48 | |

To be continued

Continued

| | | | | | | | | | | | |
|---|---|---|---|---|---|---|---|---|---|---|---|
| Meru | *R. aegyptiacus*, *H. caffer*, *Rh. fumigatus*, *Rh. landeri* *T. aegyptiaca*, *Ch. pumilus* | 20/102 | *Rh. landeri* (4), *R. aegyptiacus* (12), *H. caffer* (4) | 0/102 | | 3/270 | *R. aegyptiacus* (1), *H. caffer* (2) | 1/270 | *Rh. fumigatus* | 1/102 | *R. aegyptiacus* |
| Total | | 132/1029 | | 3/1029 | | 80/2014 | | 58/2377 | | 2/1029 | |
| Percent positive (%) | | 12.83 | | 0.29 | | 3.97 | | 2.44 | | 0.19 | |

Note: Abbreviated species names: *C. cor*; *Cardioderma cor*, *C. afra*; *Coleura afra*, *Ch. pumilus*; *Chaerophon pumiluss*, *E. helvum*; *Eidolon helvum*, *Ep. minimus*; *Epomophorous minimus*, *Ep. minor*; *Epomophorous labiatus minor*, *H. caffer*; *Hipposideros caffer*, *H. vittatus*; *Hipposideros vittatus*, *H. ruber*; *Hipposideros ruber*, *H. gigas*; *Hipposideros gigas*, *H. commersonii*; *Hipposideros commersonii*, *M. minor*; *Miniopterus minor*, *M. condylurus*; *Mops condylurus*, *My. tricolor*; *Myotis tricolor*, *O. martiensseni*; *Otomops martiensseni*, *R. aegyptiacus*; *Rousettus aegyptiacus*, *Rh. fumigatus*; *Rhinolophus fumigatus*, *Rh. eloquens*; *Rhinolophus eloquens*, *Rh. landeri*; *Rhinolophus landeri*, *Tr. affer*; *Triaenops affer*, *T. mauritiana*; *Taphozous mauritianus*, *T. aegyptiaca*; *Tadarida aegyptiaca*.

### Rotavirus

We detected two rotaviruses closely related to group A rotaviruses in two different bat species from two populations that are geographically far from each other, specifically 2980/BatRVA-*R. aegyptiacus* in Meru and 322/BatRVA-*Taphozous mauritianus* bat in Kwale region. Phylogenetic analysis based on the partial sequence of VP6 gene revealed that 2980/BatRVA was more closely related to a human RVA strain (G3P[2]) isolated from Kenya in 1997 (Ghosh et al., 2011), while 322/BatRVA was closely related to a human RVA strain (G6P[14]) isolated in Belgium in 1997 (Matthijnssens et al., 2008) (Figure 2). Based on RotaC analysis, the genotypes for the VP6 of 322/BatRVA and 2890/Bat RVA were I2 and I16 respectively. On sequence similarity comparison, the partial sequences of 2980/BatRVA and 322/BatRVA were seen to have nucleotide sequence similarities ranging from 69.67 to 87.6% and 69.07 to 94.68% with selected representative sequences of human and animal rotavirus A. The two sequences shared a 79.59% nucleotide similarity to each other and 79.86% and 79.25% nt similarity to the previously reported *Eidolon helvum*-derived RVA-KE4852/07 from Kenya (Supplementary Table S2).

### Coronavirus

Coronavirus RNA was detected in 3.97% of samples (80 out of 2,014) tested and were present in all the sampled provinces. To determine virus diversity, we carried out a phylogenetic analysis based on a 440 bp fragment of the RNA-dependent RNA-polymerase (*RdRp*) gene region. Figure 3 shows a maximum likelihood phylogeny of our CoV sequences reconstructed with other previously identified CoVs from animals and humans from the GenBank. The BtCoVs clustered within the alphacoronavirus and betacoronavirus groups. In the alphaco ronavirus group, the viruses detected in this study were mapped into 6 clusters five of which had host restriction and the other one had mixed host species. The cluster with mixed host species included *Rhinolophus* and *Hipposideros* bats, two genera that previously were classified within the same family (Teeling et al., 2005). In the betacoronavirus group, two of the clusters were HKU-9 related CoVs while the third formed a branch related to the SARS-L CoVs denoting it as a distant relative of severe acute respiratory syndrome (SARS) CoV.

### Calicivirus

Novel strains of caliciviruses were detected in two sites; Kilifi and Marsabit that are geographically distant and ecologically different, in two bat genera; *Mops* species and *R. aegyptiacus*. The sequences were identified as sapoviruses on the basis of Blastx analysis of the protein database. Two sequences derived from *M. condylurus* bats (2228/BtCalV/*Mops* and 2255/BtCalV/*Mops*) shared 100% amino acid sequence similarity and a 57.77% amino acid sequence similarity with the 22/BtCalV/*R. aegyptiacus* (Supplementary Table S3). Viruses (2228/BtCalV/*Mops* and 2255/BtCalV/*Mops* were identical sequences originating from the same colony site. These two sequences from *Mops* spp had 21.11%–64.44% nucleotide sequence identity to other members of the sapovirus group available in the GenBank while sample 22/*R. aegyptiacus*/BtCalV had 26.66%–55.55% sequence similarity. Upon phylogenetic analysis, the three BtCalVs detected in this study clustered with other reported bat sapoviruses; however, 22/*R. aegyp tiacus*/BtCalV formed an independent branch, indicating higher variability (Figure 4).

### Astrovirus

A total of 132 samples tested positive for astroviruses (12.83% positive rate) with two samples from *E. helvum* bat having co-infection with two variant strains. The sequences revealed a noteworthy viral diversity in the astroviruses present in bats in Kenya. All bat taxa scr-

Figure 2. Phylogenetic tree of the *Rotavirus* genus and the novel bat-rotaviruses detected. A maximum likelihood tree of rotaviruses group A based on partial (776bp) VP6 gene. *Porcine Rotavirus* C (Accession no. KC164679.1) was used as an outgroup. Detected bat rotavirus strains are depicted in boldface and identified with diamond node markers (◆) while other bat-derived rotaviruses are depicted by circular node marker (○). Alignments were performed using ClustalW and bootstrap values were determined using 1000 replicates on Mega5. Detected taxa are named in the following pattern: Virus strain/Identification code/Bat Host species/Country/Specific collection location/Year of collection.

eened in this study were found to harbor astroviruses except *Epomophorus* species, an observation that may be attributed to low sample numbers for this group. To the best of our knowledge this is the first reporting of bat astroviruses in Kenya as well as first detection of astroviruses in *E. helvum*, *R. aegyptiacus*, *Cardioderma cor*, *Coleura afra*, *Chaerophon pumilis*, *Triaenops affer* and *M. condylurus*. A phylogenetic analysis of the partial RdRp gene (350 nt) showed that the viral sequences detected in the Kenyan bats clustered together with the other reported bat astroviruses (Supplementary Figure S1) from Asia and Europe (Chu et al., 2008; Drexler et al., 2011; Hu et al., 2014; Kemenesi et al., 2014). The BtAstVs detected in Kenya were found to be very diverse with sequences clustering within several clades of the phylogenetic tree (Supplementary Figure S1). Further sequence comparison showed that the nucleotide identities among the Kenyan-BtAstVs ranged between 25.77 to 83.16%.

### Adenovirus

Analysis of bat derived adenovirus sequences from this study alongside those available in the GenBank clustered the 26 identified Kenyan-BtADVs within the bat *Mastadenovirus* genus (Supplementary Figure S2). The sequences occurred in 5 different clusters with cluster 1 comprising entirely of Kenyan bat-ADVs from *R. aegyptiacus*, *E. helvum*, *C. afra* and *Otomops martiensenii*.

Figure 3. Phylogenetic relationships of alpha- (A) and beta-coronaviruses (B) and viruses detected in this study. Maximum likelihood tree of α-coronaviruses and β-coronaviruses based on partial *RdRp* gene (343 bp) with 1000 bootstrap replications. Viral sequences detected in this study appear on bold face and are shown with a diamond node marker (◆). The label "(n)*" occurring after a taxon name represents clade with 'n' number of sequences collapsed for ease of visualization of the tree. Detected taxa are named in the following pattern: Virus strain/Identification code/Bat Host species/Country/Specific collection locati1on/Year of collection.

Figure 4. Phylogenetic tree of Calicivirus based on partial RdRp amino acid sequence. A maximum likelihood tree of caliciviruses based on a partial amino acid sequence. of the *RdRp* gene (91aa). The Novel bat caliciviruses detected in this study are shown in boldface and marked with a diamond (◆) on the nodes. Detected taxa are named in the following pattern: Virus strain/Identification code/Bat Host species/Country/Specific collection location/Year of collection.

Cluster 2 comprised of two sequences from *E. helvum* that formed a clade with human ADV3/E and chim panzee/Simian ADV. These two sequences were identical (100% nt identities) and had 67.14% to 71.49% nt identities with members of the phylogenetically related taxa. Clusters 3, 4 and 5 were closely related to other reported bat-derived taxa and were obtained from *Rhinolophus* species, *C. cor*, *Miniopterus* spp and *C. afra*.

### Paramyxovirus

We detected 57 paramyxovirus sequences from 5 different bat species, a positive rate of 2.4%. These viruses were found in three of the five regions sampled (Table 1), with the coastal region displaying the highest positive rates. On phylogenetic reconstruction of our sequences with other detected animal and human paramyxoviruses, our viruses clustered into three distinct groupings (Supplementary Figure S3). Cluster 1 comprised of viruses derived from different bat host species and was grouped within the Jeilong-related paramyxoviruses group. Clusters 2 and 3 comprised of viruses detected in *E. helvum* and fell within the Rubula-related and Henipa-related virus clusters. BtPMV3851, which clustered within the Henipa-related bat-PMV group, shared 97.85% nt identity with a BatPMV detected in the urine of *E. helvum* bush-meat isolated in DRC in 2009 (Accession number HE647836.1).

### Polyomavirus and Flavivirus

Polyomaviruses sequences were detected in four bat species tested; *O. martiensenni*, *E. helvum*, *Myotis tricolor* and *Miniopterus minor*. The positive rate was 1.65% much lower in comparison to the 11.8% positive rate reported by Tao *et al*. (2013) in a study of PYVs in Kenyan bats. Result of Blastn and phylogenetic analysis showed that most of the detected viruses were closely related to other reported bat polyomaviruses (Supplementary Figure S4). The virus sequences formed six separate clusters with 5 being closely related to other bat polyomaviruses and one cluster showing a distant relationship with non-human primate derived sequences.

Two flavivirus sequences were detected in *E. helvum* bats in the coastal region of Kenya. The nucleotide sequences were most closely related to Jugra and Bagaza viruses (88%–89% nt similarities) upon GenBank searches as well as phylogenetic analysis.

## DISCUSSION

Over 100 bat species (Patterson et al., 2012) exist in Kenya and occur in over a wide range of ecological regions. Due to rapid growth of human population and various anthropogenic activities, several incidences of bat colonies roosting in buildings, farms and other human inhabited areas are common. Such sites, as well as other natural bat habitats including as caves also with medium to high human activity, motivated our sampling design. Over the past few decades, about 75% of newly emerging and re-emerging pathogens largely linked to wildlife have been described (Woolhouse, 2002). Numerous viruses which in several cases occur asymptomatically in bats have been found to be of public health and veterinary health concern (Calisher et al., 2006). In several cases such as SARS-CoV, Ebola virus and Nipah virus, spillover events have resulted in disease cases in both livestock and humans (Chua et al., 2000; Liu et al., 2015; Zhong et al., 2003). Spillover from bats to humans has been shown to be direct (Van Thiel et al., 2009) or in some cases linked to availability of intermediate hosts (Wang et al., 2005).

Here we report the detection of eight viral families from bat fecal samples collected across Kenya. We detected viral sequences related to *Adenoviridae*, *Astroviridae*, *Caliciviridae*, *Coronaviridae*, *Flaviviridae*, *Paramyxoviridae*, *Polyomaviridae* and *Reoviridae* (specifically *Rotavirus* genus). The detection rates for the different viral families varied across the country with counties in the coastal region displaying the highest virus diversity (Table 1). The high virus richness in the coastal region may be attributed to a higher diversity in bat species screened. Among the detected sequences there were novel rotaviruses, caliciviruses and astroviruses. Similar to other previous studies carried out to screen these viruses in bats, we report high detection rates for AstVs compared to RVA and CalV (Hu et al., 2014; Kemenesi et al., 2014; Tse et al., 2012).

### Rotavirus

Rotavirus A sequences have previously been detected in various bat species in a number of countries including Kenya (*E. helvum*), China (*Rhinolophus hipposideros* and *Aselliscus stoliczkanus*), France (*Myotis mystacinus*) and recently in Cameroon (*E. helvum*) and Brazil (multiple species) (Asano et al., 2016; Dacheux et al., 2014; Esona et al., 2011; He et al., 2013; Xia et al., 2014; Yinda et al., 2016). Group H rotaviruses which have previously been reported in humans and pigs have also been recently detected in bats in South Korea (Kim et al., 2016). Previous studies, reported that the detected bat RVAs were potentially reassortant strains carrying genes from the human RVA hence indicating a high possibility of interactions between bat and human strains of the virus. Partial sequences of BatRVA *VP6* gene amplified in this study were found to be quite closely related to human *Rotavirus A*. Viruses 322/BatRVA and 2980/BatRVA detected from *T. mauritianus* and *R. aegyptiacus*, respectively, were most closely related to RVA-VP6 from Belgium (EF554119.1) and Kenya (HM 627557.1) which have the genotype constellations G6-P[14]-I2-R2-C2-M2-A3-N2-T6-E2-H3 and G3-P[2]-I16-R8-C5-M5-A5-N5-T5-E13-H5 respectively. The 322/BatRVA was assigned genotype I2 which has been described in several species including but not limited to cow, simian, rabbit as well as human but not in bats. VP6 genotypes described in bats are I15 in Kenya and Cameroon, I8 in China and I22 in Cameroon (Esona et al., 2010; He et al., 2013; Yinda et al., 2016). The 2980/

BatRVA was assigned I16 genotype with 87.4% nt similarity to the Kenyan human B10 isolate, which is notably close to the 85% cut-off point. The I16 genotype has only been previously assigned to the Kenyan human isolate, hence our virus is the second sequence to enter this group. This alludes to reassortment of human and bat virus strains supporting previous research findings. These two viruses were genotypically distinct from each other, as they shared a nucleotide similarity of 79.9% which is lower than the set cut-off values of 85% for *VP6* gene (Matthijnssens et al., 2008). Further sequence characterization of the virus strains detected in our study is essential to provide additional information on specific genotype. Our study provides the first report of batRVA in *T. mauritianus* and *R. aegyptiacus*. The presence of several strains of rotaviruses in multiple species of bats shows that bats are natural reservoirs of rotavirus. The alleged reassortment with human and other animal rotaviruses could indicate a strong potential of emergence and re-emergence of novel and known infectious rotaviruses.

### Coronavirus

Diverse strains of coronavirus have been described in bats. Of these strains, SARS-CoV and MERS CoV have caused substantial disease in humans. These two viruses cause acute respiratory distress syndrome (ARDS) with high mortality rates (Hu et al., 2015). SARS-CoV was first described in China in 2002 (Zhong et al., 2003) while MERS-CoV first emerged in Saudi Arabia in 2012 (Zaki et al., 2012). Since then several SARS-CoV and MERS-CoV related viruses have been discovered in other areas of the world. Our results display a high diversity of bat CoVs in the Kenyan population including the detection of 229E-related CoV and a distant relative of the SARS-CoV. The 229E-related CoVs sequences were detected in bats of *Hipposideros* species from two different sites: Taita Taveta and Meru counties that are far apart. The colonies roost in buildings within wildlife conservation areas, with a high level of human activity. On phylogenetic analysis as well as nucleotide and amino acid sequence similarity comparisons, our 229 ECoV-like sequences were more closely related to Alpaca-CoV which was first described in the USA from camelid species (Crossley et al., 2012). The sequences also show a distant relationship to other bat-derived 229E-like CoVs from Ghana (Corman et al., 2015). These finding support the hypothesis of ancestral origin of human 229E virus from bats with a camelid intermediate host.

### Calicivirus

Bat caliciviruses have previously been reported in *Hipposideros pomona* bats from China (Tse et al., 2012), *Myotis daubentonii*, *Myotis alcuthoe* and *Eptesicus serotonus* from Hungary (Kemenesi et al., 2014). Here, we found Sapovirus sequences in two different species (*Mops condylurus* and *Rousettus aegyptiacus*) which have not been previously reported to harbor caliciviruses. The viruses were detected within the same province (Former Eastern province) but from two sites (over 300KM apart) that vary in ecology, one being a cave and the other a household attic. Sequence comparison and phylogenetic analyses show the detected sequences to be divergent from previously reported BtCalVs. The viruses from *Mops* species segregated with other reported bat CalVs but were genetic variants with 21.11–64.44% nucleotide sequence similarities with other reported bat sapoviruses. The virus sequence derived from *R. aegyptiacus* species which was more genetically distant formed an independent branch related to the bat sapovirus cluster. To our knowledge, our results are the first documented report of BatCalVs in Kenya and Africa. Given that we obtained the viral sequences from two species in separate sites, there is a high likelihood that more strains of CalVs are circulating in bat populations.

### Astrovirus

The prevalence and diversity of astroviruses in the Kenyan bat population was high, recording the highest detection rate and most widely distributed virus species (by location and host species) compared to all others detected in this study. The diversity was reflected in both within host species and site-origin of the viruses. Phylogenetic analysis based on partial *RdRp* gene revealed a significantly high diversity among the detected strains of BtAstVs (Supplementary Figure S1). Unlike previous reports (Hu et al., 2014), the strains of viruses present in the Kenyan bats exhibit only partial host restrictions with clades having sequences from various bat species. Sequences of BtAstVs from *E. helvum* and *R. aegyptiacus* irrespective of location of origin, display a close relatedness between strains in comparison to sequences derived from other bat species. Further phylogenetic comparison of our BtAstV sequences with other non-bat derived astroviruses reveal a distant relationship of *R. aegyptiacus* derived sequences to porcine AstVs and *E. helvum* derived sequences to feline astroviruses, while the other species remain clustered with bat-derived AstVs. Nucleotide identities of *R. aegyptiacus* to porcine AstVs ranges from 60.13–64.26% and *E. helvum* to feline AstVs ranges from 51.20–70.44%, values which are higher than similar comparisons to BatAstVs (Data not shown).

### Adenovirus

A previous study reported BtADVs in *Chaerophon* and *Otomops* bat species in Kenya (Conrardy et al., 2014). We detected BtADVs in 3 additional species; *M. minor*.

*M. tricolor*, *E. helvum* as well as in *O. martiansenii*. The BtADVs mapped to 5 clusters, all four of which were within clades containing other BtADVs (Supplementary Figure S2). One of the clusters containing two BtADVs from *E. helvum* species, infers a distant relationship to human ADVs and simian ADV. This is consistent with results obtained upon nucleotide sequence comparison on NCBI (BAT062/BtADV 89% nt identity to human ADV-E on Blastn and 69% identities to Simian ADV on Blastx; while BAT085/BtADV had 85%nt identity to Simian ADV3 on Blastn and 69% to simian ADV on Blastx). These two *E. helvum* derived ADV sequences share 95.5% nucleotide identity.

### Paramyxovirus

Paramyxoviruses have previously been detected in five bat species in Kenya including; *C. cor*, *Chaerophon pumulis*, *Miniopterus* species, *O. martiensseni* and *R. aegyptiacus* (Conrardy et al., 2014). Our results indicated a wider host range with detection of viral sequences in six additional bat species including; *E. helvum*, *T. mauritianus*, *Hipposideros* species, *C. afra*, *M. condylurus* and *Rhinolophus* species. Consistent with the previous study, *O. martiensenii* displayed the highest detection rate of PMVs. In both studies, Henipa-related viruses were detected in fruit bats; in *E. helvum* in our study and *R. aegyptiacus* in the study by Conrardy *et al*. (2014). These two separate studies report presence of Henipa-related viruses in fecal swabs of fruit bats in Kenya. The *E. helvum* colony found positive for this virus roosted within a highly populated market place and may pose a public health risk. Henipaviruses have been reported to cause serious disease in humans and have a high mortality rate of 70% (Aljofan, 2015). Detection of Henipa-related virus in *E. helvum* and *R. aegyptiacus*

### Polyomavirus and Flavivirus

The PYVs detected in this study were seen to be Orthopolyomaviruses based on Blastn and phylogenetic analysis. Similar to a previous study, further analysis of the partial VP1 gene of the detected PYVs showed relatedness to bat PYVs available in the GenBank, as well as to non-human primate PYVs (Tao et al., 2013). These results infer a historical common ancestor for the polyomaviruses bat and non-human primates.

Interestingly, flavivirus sequences were also detected in the *E. helvum* colony roosting near a market. These two flavivirus sequences were nearly identical, bearing 93.58% nucleotide sequence identity. The sequences had 83.33% and 87.17% nt sequence identity with their closest characterized flavivirus relative; Jugra virus (DQ 859066.1). Jugra virus which has previously been detected in *Cynopterus brachiotis* (lesser short-nosed fruit bat) is a mosquito transmissible virus and a distant relative of Dengue virus (Calisher et al., 2006). Dengue virus disease is endemic in East Africa, including Kenya (Sutherland et al., 2011). Further characterization of these detected virus strains is important to understand their genome as well as their relationship to other disease-causing flaviviruses such as Dengue virus.

Detection of viruses in bat fecal material is of high significance in terms of public health as it is one of the most direct ways that bat pathogens can be transmitted to humans. For instance, touching bat feces and surfaces contaminated with bat feces is a risk for human infection especially if skin on hands are broken. Fecal droppings from fruit bats may contaminate fruits on trees and vegetables, hence consumption of raw or unwashed fruits and vegetables may be a health risk. Specifically, rotavirus, calicivirus and astroviruses which are transmitted via the oral-fecal route and cause significant health burden in the third world nations. The high similarity of the bat RVA to the human strain of the virus raises genuine concerns of possible transmission considering the cohabitation of humans and many bat species. Our results provide new and extended knowledge with regards to the virus diversity in Kenyan bat species. We recommend sustained surveillance to provide status of most current circulating pathogens and hence inform preparedness in cases of bat-related emerging and re-emerging disease outbreaks. The role of bats in maintenance and circulation of the detected novel viruses (astro, calici- and rotaviruses) is still largely unknown and further studies ought to be carried out to this end.

### ACKNOWLEDGMENTS

We are grateful for the Government of Kenya who permitted this study, specifically the Directors of Kenya Wildlife Service and National Museums of Kenya. We also appreciate the field and laboratory teams in Kenya and China who assisted in many different ways towards the success of this work. This work was funded by Sino-Africa Joint Research Center (SAJC201313 and SAJC 201605).

### COMPLIANCE WITH ETHICS AND GUIDELINES

The authors declare they have no conflicts of interest. The study was approved by the Kenya Wildlife Service, research permit, KWS/BRM/5001. All institutional and National guidelines for care and handling use of animals were followed.

### AUTHOR CONTRIBUTIONS

Z-LS, SO and CW designed the study. Z-LS, SO, BA and VO coordinated the study. Z-LS, SO, VO, BA, FG,

XYG, XLY, LJW and CW participated and supported in field sampling. CW and AZ carried out molecular studies under the supervision of HB. CW analyzed the data. CW and Z-LS drafted the manuscript. All authors read and approved the final manuscript

Supplementary figures/tables are available on the websites of *Virologica Sinica*: www.virosin.org; link.springer.com/journal/12250.

### OPEN ACCESS

This article is distributed under the terms of the Creative Commons Attribution 4.0 International License (https://creativecommons.org/licenses/by/4.0/), which permits unrestricted use, distribution, and reproduction in any medium, provided you give appropriate credit to the original author(s) and the source, provide a link to the Creative Commons license, and indicate if changes were made.

## Electronic supplementary material

Molecular detection of viruses in Kenyan bats and discovery of novel astroviruses, caliciviruses and rotaviruses
