# Supplementary material for: Molecular detection of viruses in Kenyan bats and discovery of novel astroviruses, caliciviruses and rotaviruses
Source: Virol Sin. 2017 Apr 6;32(2):101–14. doi: 10.1007/s12250-016-3930-2 (PMC6702250; doi:10.1007/s12250-016-3930-2)
Supplement: Supplementary file 1 — Molecular detection of viruses in Kenyan bats and discovery of novel astroviruses, caliciviruses and rotaviruses [file 12250_2016_3930_MOESM1_ESM.pdf]

Electronic Supplementary Material

Molecular detection of viruses in Kenyan bats and discovery of novel astroviruses, caliciviruses and rotaviruses

Cecilia Waruhiu[1,2], Sheila Ommeh[3]✉, Vincent Obanda[4], Bernard Agwanda[5], Francis Gakuya[4], Xing-Yi Ge[1,2], Xing-Lou Yang[1,2], Li-Jun Wu[1], Ali Zohaib[1], Ben Hu[1,2], Zheng-Li Shi[1,2]✉

1. Key Lab of Special Pathogens, Wuhan Institute of Virology, Chinese Academy of Sciences, Wuhan 430071, China.
2. Sino-Africa Joint Research Center, Chinese Academy of Sciences, Wuhan 430071, China.
3. Institute of Biotechnology Research, Jomo Kenyatta University of Science and Technology, Nairobi 00200, Kenya.
4. Veterinary Services Department, Kenya Wildlife Service, Nairobi 00100, Kenya.
5. Mammalogy Section, National Museum of Kenya, Nairobi 00100, Kenya.

Supporting information to DOI: 10.1007/s12250-016-3930-2

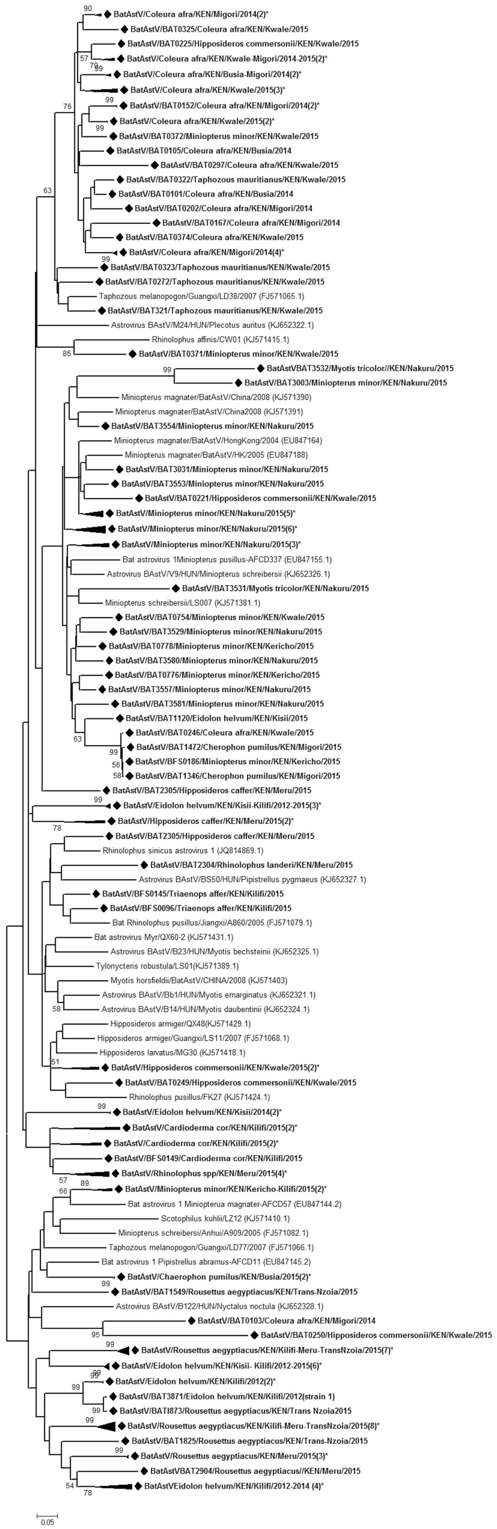

Figure S1. Phylogenetic tree of astroviruses. Phylogenetic tree of bat-astroviruses based on partial *RdRp* gene sequence (350 nt). The BtAstVs detected in this study are shown in boldface and denoted with a diamond node marker (◆). Taxon labels containing the label "(n)*" represent a clade with 'n' number of sequences collapsed for ease of visualization of the tree. Detected taxa are named in the following pattern: Virus strain/Identification code/Bat Host species/Country/Specific collection location/Year of collection.

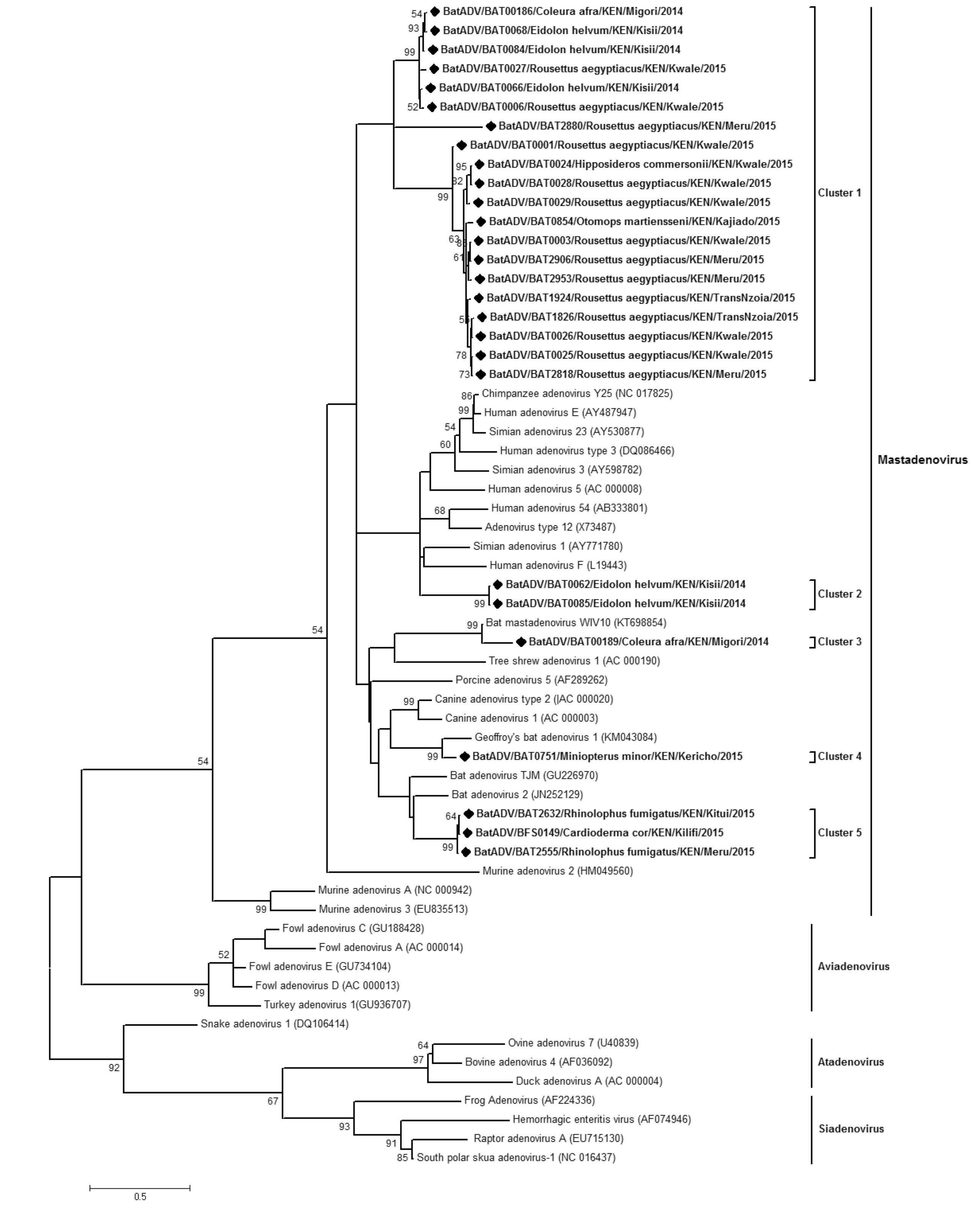

Figure S2. Phylogenetic tree of adenoviruses. Maximum likelihood tree of adenoviruses based on partial polymerase gene sequence (207 nt). The bat adenoviruses detected in this study are shown in boldface and denoted with a diamond (◆) on the node marker. Detected taxa are named in the following pattern: Virus strain/Identification code/Bat Host species/Country/Specific collection location/Year of collection. All ADVs detected in this study were found to be Mastadenoviruses.

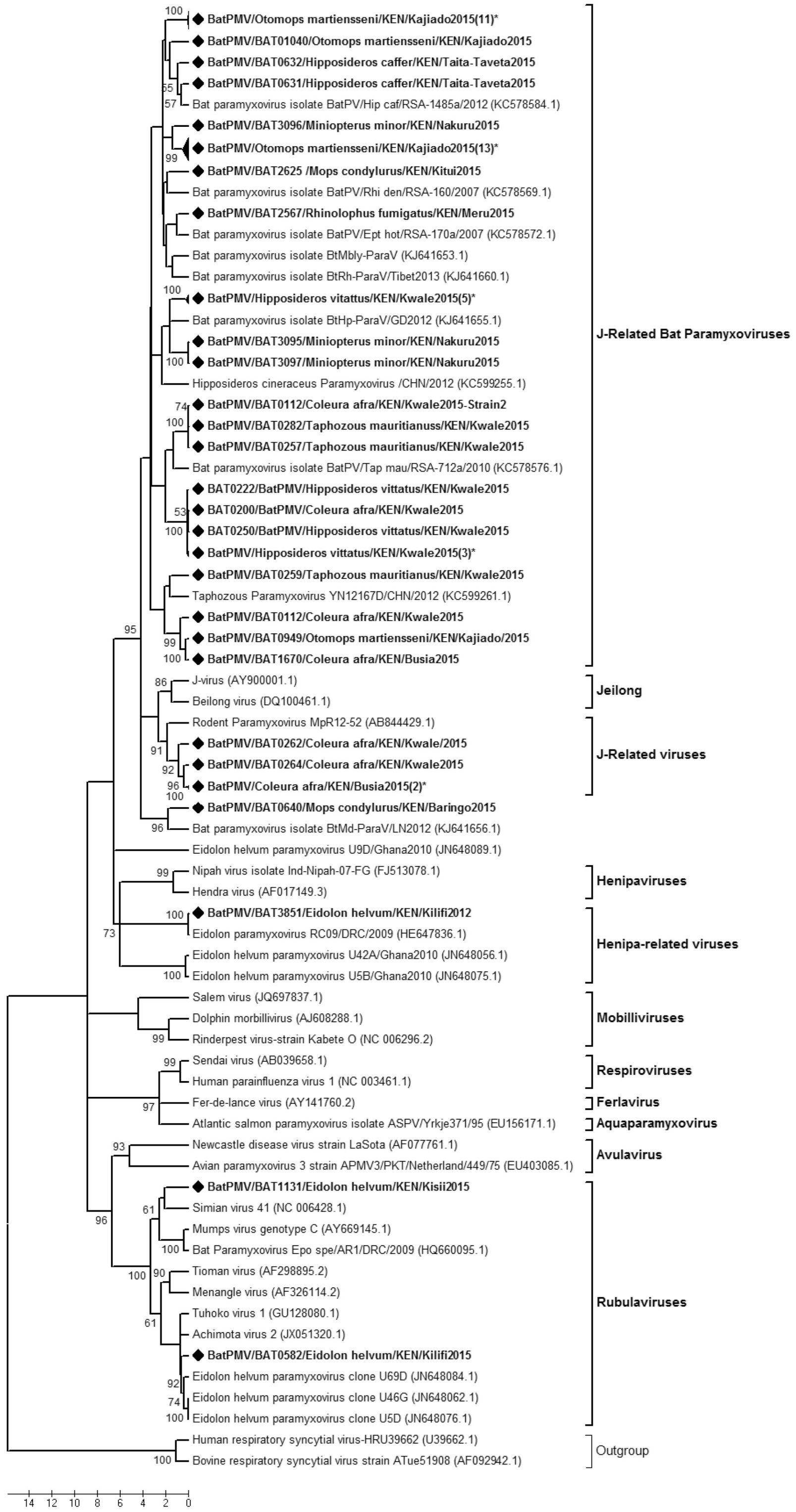

Figure S3. Phylogenetic tree of paramyxovirus. Maximum likelihood tree of paramyxoviruses based on partial polymerase gene sequence (409 nt). The BtPMVs detected in this study are shown in boldface and denoted with a diamond (◆) on the node marker. Taxon labels containing the label "(n)*" represent a clade with 'n' number of sequences collapsed for ease of visualization of the tree. Detected taxa are named in the following pattern: Virus strain/Identification code/Bat Host species/Country/Specific collection location/Year of collection.

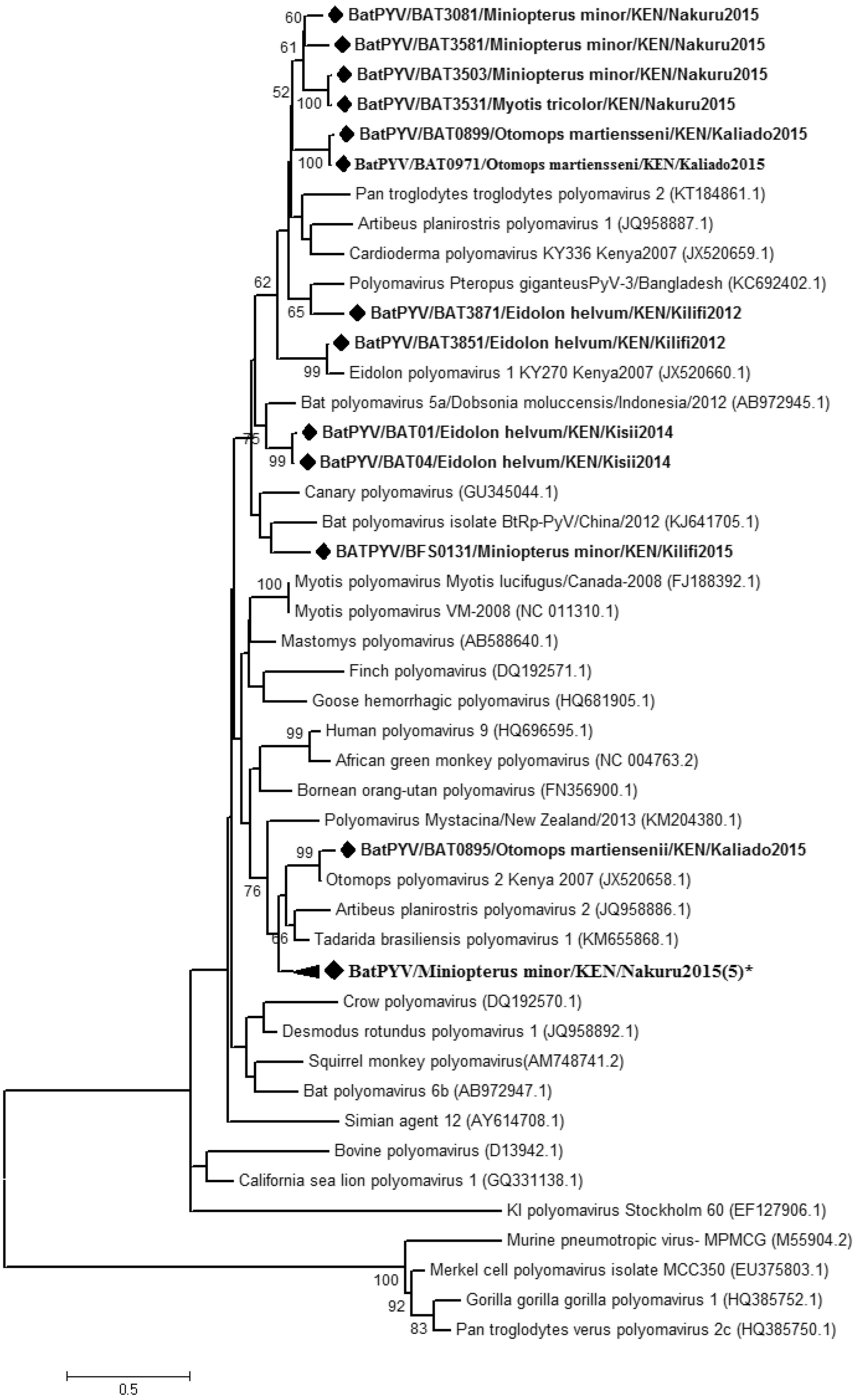

Figure S4. Phylogenetic tree of polyomavirus. Phylogenetic tree of polyomaviruses based on partial *VP1* gene (236 bp). The BtPYVs detected in this study are shown in boldface and denoted with a diamond node marker (◆). Taxon labels containing the label "(n)*" represent a clade with 'n' number of sequences collapsed for ease of visualization of the tree. Detected taxa are named in the following pattern: Virus strain/Identification code/Bat Host species/Country/Specific collection location/Year of collection.

Table S1. Table showing the primer sets used in the study

Target virus name		Primer name	Primer sequence (5'–3')	Final amplicon size (nt)	Reference
Adenovirus	Round 1	POLF1	CAGCCKCKQTTRTGYAGGGT	250 bp	Li Yan *et al.*, 2010
		POLR1	GCHACCATYAGCTCCAACTC		
	Round 2	POLF2	GGGCTCRTTRGTCCAGCA		
		POLR2	TAYGACATCTGYGGCATGTA		
Astrovirus	Round 1	AstroFWD1	GARTTYGATTGGRCKCGKTAYGA	422 bp	Chu *et al.*, 2008
		AstroFWD2	GARTTYGATTGGRCKAGGTAYGA		
		AstroRVS1	GGYTTKACCCACATNCCRAA		
	Round 2	AstroFWD3	CGKTAYGATGGKACKATHCC		
		AstroFWD4	AGGTAYGATGGKACKATH CC		
		AstroRVS1	GGYTTKACCCACATNCCRAA		
Calcivirus		Cal-P289	TGACAATGTAATCATCACCATA	319-331bp	Jiang *et al.*, 1999
		Cal-P290	GATTACTCCAAGTGGGACTCCAC		
Coronavirus	Round 1	CoV-FWD3	GGTTGGGAYTAYCCHAARTGTGA		
		CoV-RVS3	CCATCATCASWYRAATCATCATA	440 bp	Modified from Watanabe *et al.*, 2010
	Round 2	CoV-FWD4/ Bat	GAYTAYCCHAARTGTGAYAGAGC		
		CoV-RVS3	CCATCATCASWYRAATCATCATA		
Flavirirus	Round 1	Flavi1+	GAYYTIGGITGYGGIIGIGGIRGITGG		
		Flavi1–	TCCCAICCIGCIRTRTCRTCIGC		
	Round 2	Flavi2+	YGYRTIYAYAWCAYSATGGG	141 bp	Sanchez-seco *et al.*, 2005
		Flavi2–	CCARTGITCYKYRTTIAIRAAICC		
Filovirus	Round 1	FiloNPF1	TGGCARTCRGTIGGACAYATGATGGT	391 bp	In- house designed
		FiloNPF2	TGGCTYACYACAGGYCAYATGAAAGT		
		FiloNPR1	TRATYTCRTTYTTITTCTGITGGAA		
		FiloNPR2	TGATCTCATTTTTCCGGGAGTGGAA		
		FiloNPR3	TGATYTCAGTYTTYTGAAGITGGAA		
	Round 2	FiloNPF3	TGGTIGCIGGICAYGATGCIAAYGA		
		FiloNPF4	TGGTGACAGGTCATGATGCMTATGA		
		FiloNPR4	TCAGCYTCAGTAGCAGCCTCAC		
		FiloNPR5	TCRGCYTCAGTKGCWGCITCTC		
		FiloNPR6	TCYGCATCATGTGCIGCCTCTC		
Paramyxovirus	Round 1	PAR-F1	GAAGGITATTGTCAIAARNTNTGGAC	561 bp	Tong *et al.*, 2008
		PAR-R	GCTGAAGTTACIGGITCICCDATRTTNC		
	Round 2	PAR-F2	GTTGCTTCAATGGTTCARGGNGAYAA		
		PAR-R	GCTGAAGTTACIGGITCICCDATRTTNC		
Polyomavirus	Round 1	VP1/lf	CCAGACCCAACTARRAATGARAA	249–273 bp	Johne *et al.*, 2005
		VP1/1r	AACAAGAGACACAAATNTTTCCNCC		
	Round 2	VP1/2f	ATGAAAATGGGGTTGGCCCNCTNTGY AARG		
		VP1/2r	CCCTCATAAACCCGAACYTCYTCHAC YTG		
Rotavirus	Round 1	VP6-F	GACGGVGCRACTACATGGT	1356 bp	Matthijnssens *et al.*, 2006
			GTCCAATTCATNCCTGGTGG		

Table S2. Nucleotide sequence identity comparison for members of the Rotavirus A group

	322BatRVA/*T. affer*/KEN/2015	2980BatRVA/*R. aegyptiacus*/KEN//2015
	Percentage nt similarities (%)	
**322BatRVA/*Taphozous affer*/KEN/Kwale/2015**		
**2980BatRVA/*Rousettus aegyptiacus*/KEN/Meru/2015**	79.59	
GU983675.1|_Rotavirus_A_bat/4852/Kenya/2007	79.86	79.25
KX268791.1|_Rotavirus_A_isolate_Bat-wt/CMR	74.01	73.75
KX268780.1|_Rotavirus_A_isolate_Bat-wt/CMR	80.12	79.59
KX268769.1|_Rotavirus_A_isolate_Bat-wt/CMR	74.1	73.84
KX268758.1|_Rotavirus_A_isolate_Bat-wt/CMR	73.84	73.75
KX268747.1|_Rotavirus_A_isolate_Bat-wt/CMR	73.84	73.75
KJ020894.1|_Rotavirus_A_isolate_RVA/Bat-tc/CHN	79.51	81.16
HM627557.1|_Human_rotavirus_A_strain_B10	80.64	87.61
EF554119.1|_Rotavirus_A_strain_RVA/Human-wt/BEL	94.68	80.12
KP882661.1|_Rotavirus_A_strain_RVA/Human-wt/GHA	94.42	80.03
KP882738.1|_Rotavirus_A_strain_RVA/Human-wt/KEN	79.16	79.59
K02086.1|RO2SEG6_Human_Wa_rotavirus	79.25	78.11
AY787645.1|_Human_rotavirus_A_strain_TB-Chen	85.52	79.77
DQ146664.1|_Rotavirus_A_strain/Human-wt/BGD/Dhaka12	79.33	79.33
DQ146702.1|_Rotavirus_A_strain_RVA/Human-tc/THA	79.94	81.95
DQ490555.1|_Rotavirus_A_strain_RVA/Human-wt/BGD	92.15	81.16
DQ870507.1|_Rotavirus_A_strain_RVA/Human-tc/USA	86.13	80.03
EF554086.1|_Rotavirus_A_strain_RVA/Human-wt/BEL	91.8	80.9
EF583048.1|_Rotavirus_A_strain_RVA/Human-tc/GBR	78.63	78.55
AY594670.1|_Rotavirus_strain_TUCH-Rhesus	80.73	81.95
FJ422136.1|_Rotavirus_A_strain_RVA/Rhesus-tc/USA	93.11	80.29
X69487.1|_Rotavirus_A_Porcime strain YM	79.68	77.76
KP753064.1|_Rotavirus_A_strain_RVA/Pig-wt/ZAF	93.19	80.55
AF317123.1|_Porcine_rotavirus	79.33	76.89
KJ752065.1|_Rotavirus_A_strain_RVA/Cow-wt/ZAF	94.42	79.94
GU384194.1|_Bovine_rotavirus_A_isolate_DQ-75	93.28	79.59
FJ495131.1|_Rotavirus_A_strain_RVA/Antelope-wt/ZAF	94.5	79.94
FJ347126.1|_Rotavirus_A_strain_RVA/Guanaco-wt/ARG	91.89	80.2
D82970.1|RO1ICPVP6A_Chicken Rotavirus A	69.31	67.91
D16329.2|AROVP6_Avian_rotavirus_A	70.18	70.09
U65988.1|MRU65988_Murine_rotavirus A	79.07	78.11
AB971764.1|_Rotavirus_A_strain:RVA/SugarGlider-tc/JPN	78.46	80.38

Table S3. Percentage amino acid sequence similarity of detected BtCalV with other representative caliciviruses

	CalV22/*R. aegyptiacus*/ KEN/Klf/2015	CalV2228/*Mops condylurus*/KEN/Mars/ 2015	CalV2255/*Mops condylurus*/KEN/Mars/ 2015
	Percentage nt similarities (%)		
**CalV22/*R. aeg*/KEN/Klf/2015**			
**CalV2228/*Mops*/KEN/Mar/2015**	68.13		
**CalV2255/*Mops*/KEN/Mar/2015**	68.13	100	
AAA47285.1|Rabbit Hemorrhagic DV	61.53	51.64	51.64
AAB50465.1|Norwalk Virus	41.75	40.65	40.65
AAA92983.1|Southampton virus	41.75	40.65	40.65
CAA93445.1|European brown hare syndrome virus	61.53	52.74	52.74
AAB97767.2|Hawaii Calicivirus	46.15	49.45	49.45
AAA96501.2|San Miguel sea lion virus	54.94	49.45	49.45
AAL99277.1|Calicivirus NB	52.74	49.45	49.45
AAT39864.1|Porcine enteric CalV	60.43	64.83	64.83
AAY60849.1|Nebovirus Newbury 1	52.74	50.54	50.54
ABD16233.1|Porcine sapovirus	65.93	71.42	71.42
ABO43773.1|Porcine enteric sapovirus	65.93	70.32	70.32
ACB38131.1|Tulane virus	38.46	34.06	34.06
ACJ63217.1|Pig sapoviris/NLD	53.84	56.04	56.04
ACQ44561.1|Pig calicivirus/CAN	38.46	38.46	38.46
ACQ44563.1|Pig calicivirus/CAN	38.46	38.46	38.46
ACZ69384.1|Swine sapovirus/BR	50.54	52.74	52.74
AFH89833.1|Turkey calicivirus	54.94	50.54	50.54
AFH89835.1|Chicken calicivirus	53.84	48.35	48.35
AFJ39353.1|Bat sapovirus TLC39/HK	68.13	74.72	74.72
AFJ39355.1|Bat sapovirus TLC58/HK	68.13	74.72	74.72
AFM93994.1|Recovirus/Bangladesh	43.95	39.56	39.56
AID54988.1|BatCalV/M63/HUN/2013	67.03	78.02	78.02
AID54989.1|Bat Calicivirus/HUN	32.96	27.47	27.47
AJA31687.1|Porcine sapovirus/ETH	61.53	67.03	67.03
AJA31689.1|Porcine sapovirus/ETH	40.65	42.85	42.85
AGH15844.2|Swine sapovirus/USA	57.14	57.14	57.14
